# Time management practice and associated factors among health professionals working in public and private hospitals in Dessie City, Northeast Ethiopia: a comparative cross-sectional study

**DOI:** 10.3389/fpsyg.2024.1366457

**Published:** 2024-05-01

**Authors:** Ali Yimer, Amsalu Feleke, Lake Yazachew, Mohammed Ahmed, Seada Seid, Mohammed Adem, Semira Muhidin Mustofa, Merkineh Markos, Nurye Sirage, Abdulaziz Kebede Kassaw, Nuru Muhammed Mustefa, Dawed Ali, Endalkachew Dellie

**Affiliations:** ^1^Department of Public Health, College of Health Sciences, Woldia University, Woldia, Ethiopia; ^2^Department of Health Systems and Policy, Institute of Public Health, College of Medicine and Health Sciences, University of Gondar, Gondar, Ethiopia; ^3^School of Medicine, College of Health Sciences, Woldia University, Woldia, Ethiopia; ^4^Department of Optometry, School of Medicine, College of Health Sciences, University of Gondar, Gondar, Ethiopia; ^5^Department of Midwifery, College of Health Sciences, Woldia University, Woldia, Ethiopia; ^6^Department of Health Informatics, College of Medicine and Health Sciences, Wollo University, Dessie, Ethiopia; ^7^Department of Statistics, College of Natural and Computational Sciences, Woldia University, Woldia, Ethiopia; ^8^Department of Psychiatry, College of Medicine and Health Sciences, University of Gondar, Gondar, Ethiopia

**Keywords:** time management practice, health professionals, public and private hospitals, Dessie City, Northeast Ethiopia

## Abstract

**Background:**

Time management is a widely ignored concern in all types of organizations, including the healthcare system, despite its crucial role in the achievement of personal and organizational goals. Therefore, determining the practice of time management and its associated factors among health professionals is of paramount importance.

**Objective:**

We aimed to assess time management practice and associated factors among health professionals working in public and private hospitals in Dessie City, Northeast Ethiopia.

**Methods:**

A comparative cross-sectional study was conducted from 24 March to 24 April 24 2021 among 660 health professionals (220 from private hospitals and 440 from public hospitals), who were randomly selected using a stratified sampling technique. A self-administered questionnaire was used to collect data. A multivariable binary logistic regression model was run separately for public and private hospitals. A *p*-value of < 0.05 and a 95% confidence interval were reported to be statistically significant.

**Results:**

A total of 615 participants responded to the survey, which resulted in a response rate of 93.2%. The magnitude of good time management practice among health professionals in both public and private hospitals was reported to be 57.1% (95% CI: 53.5–60.8%). However, the magnitude differed between public [50.1% (95% CI:45.5–54.7%)] and private hospitals [70.9% (95% CI: 63.5–76.7%)]. Living with families (AOR: 5.39, 95% CI: 1.84–15.77), satisfaction with compensation and benefits (AOR: 7.83, 95% CI: 1.97–31.16), satisfaction with work autonomy (AOR: 7.10, 95% CI: 1.94–25.95), and having a good plan (AOR: 3.42, 95% CI: 1.15–10.13) were statistically significant predictors of time management practice in private hospitals. Satisfaction with an organizational policy and strategy (AOR: 2.34, 95% CI: 1.25–4.36), performance appraisal (AOR: 1.95, 95% CI: 1.13–3.36), work autonomy (AOR: 1.92, 95% CI: 1.12–3.27), and the good approach of employees toward time (AOR: 2.12, 95% CI: 1.26–3.58) were statistically significant predictors of time management practice in public hospitals.

**Conclusion:**

The magnitude of a good time management practice in public and private hospitals in the study area was low. The practice was observed to be higher in private hospitals than in public hospitals. The study revealed that the magnitude and associated factors of time management practice vary between hospitals, indicating the need for targeted intervention.

## Introduction

Time is a non-renewable resource that is essential for all activities and processes in an organization (Rosemary et al., 1992; Ojokuku and Obasan, 2011). When people have more tasks to do and do not have enough time, they explain that lack of time is the reason for their unsuccessful goals, undesired performance, and poor productivity (Adeojo, 2012). However, time management emphasizes the principle that it is more relevant to do the right things than to do things right (Akintayo et al., 2020).

A fraction of a microsecond is vital for health professionals because they address the health needs of the patient and society at large (Porta et al., 2013; Gordon and Borkan, 2014; Said, 2014). Time management skills enable health professionals to be disciplined at work and obtain professional satisfaction, growth, and achievement (Rosemary et al., 1992; Gordon and Borkan, 2014). Consistent with this notion, effective time management practice (TMP) is a base for implementing sound management practices, which are building blocks of the health system, and implementing health policies (Benowitz, 2001; World Health Organization, 2010; Health Mo, 2017).

The magnitude of TMP differs between countries; for example, the magnitude of TMP is 69.5% in Palestine, 49% in Iran, 53.7% in Ghana, and 56.4% in Ethiopia (Qteat and Sayej, 2014; Meiring, 2017; Asiedu, 2019; Chanie et al., 2020). Poor TMP leads to uncontrollable stress, poor work quality, inefficient workflow, poor professional reputation, and a stalled career (Meiring, 2017). Various factors influence TMP, including personal-related factors (planning, socialization, and employee attitude toward time) and job satisfaction factors (work environment, co-worker relationship, workload, performance evaluation, organizational policy and strategy, compensation and benefit, work autonomy, supervisor support, professional development, and recognition and reward; Olowookere et al., 2015; Abdulwadod, 2017; Nigussie and Injibara, 2019; Chanie et al., 2020). Furthermore, factors including salary, educational status, age, type of profession, marital status, type of institution, and work unit affect TMP (Qteat and Sayej, 2014; Olowookere et al., 2015; Nigussie and Injibara, 2019). Different organizations have different TMP trends, which resulted in a discrepancy in achieving productivity and successful performance of employees (Chanie et al., 2020). Poor TMP in a health institution is a neglected problem, as it is the bottleneck to the success of employees and the organization (Chanie et al., 2020). Previous researchers reported TMP among health professionals in public health sectors (Qteat and Sayej, 2014; Nigussie and Injibara, 2019; Chanie et al., 2020). However, TMP also affects private health institutions, which remain neglected. Thus, the assessment of TMP in a public vs. private institutional setting would be vital. Furthermore, this study extensively addressed the TMP by incorporating variables that were not addressed in previous studies such as socialization, work autonomy, workload, employee's approach toward time, profession, living conditions, coworker relationship, professional development, and working unit (Qteat and Sayej, 2014; Nigussie and Injibara, 2019; Chanie et al., 2020). Given the identified gap in the literature, this study examined the magnitude of time management practice and identified associated factors among health professionals working in public and private hospitals in Dessie City, Ethiopia, to understand the size of the problem and identify key areas to tailor an effective and targeted intervention.

## Materials and methods

### Study design and area

An institutional-based comparative cross-sectional study was conducted from 24 March to 24 April 2021 in public and private hospitals in Dessie City, which is located 401 km from Addis Ababa, the capital of Ethiopia. The city comprises two public hospitals, eight public health centers, and five general private hospitals serving more than 8 million people in the area. The study focused on public and private hospitals to obtain a relatively similar study population.

### Source populations

The source populations of the study were all health professionals working in public and private hospitals in Dessie City.

### Study populations

The study populations included all selected health professionals who were working in public and private hospitals in Dessie City and who met the inclusion criteria.

### Eligibility criteria

#### Inclusion criteria

The study included all full-time health professionals employed in public and private hospitals in Dessie City who were available during the study period.

#### Exclusion criteria

The study excluded those health professionals who served for < 6 months.

Health professionals belonging to environmental health and health informatics institutions and others who were employed only in either institution but absent in private or public hospitals were excluded.

### Sample size determination

The sample size was determined using Epi info software assuming health professional planning as a factor affecting time management practice, which was obtained from a previous study (Chanie et al., 2020), an odds ratio (OR) of 2.86, a ratio of 2 for public vs. private institutions, 61.33% planning in the unexposed group at the 95% confidence level, 95% power, and 10% for the non-response rate. The maximum sample size for the study was 660 (220 health professionals from private hospitals and 440 health professionals from public hospitals).

## Sampling procedure

The samples were selected from seven departments using a stratified sampling technique using the profession as a stratum. To select 660 health professionals from private and public hospitals, the payroll of each health professional from the human resource management database in the hospital was used as a sampling frame for each professional category, and then the number of employees in each profession was proportionally allocated to the sample size. The frame contains 937 health professionals (675 from public hospitals and 262 from private hospitals). Finally, the study subjects from each profession were selected by a simple random sampling technique.

### Study variables

The outcome of interest was TMP, which was defined as working smart by using time effectively at the work site. It was measured by 12 items, and each was scored with a 5-point Likert scale, with 1 indicating strongly disagree and 5 indicating strongly agree. After the cutoff point was calculated using the demarcation threshold formula $\left(\;\frac{\text{Total}\;\text{highest}\;\text{score - Total}\;\text{lowest}\;\text{score}}{2}\right)\;+total\;lowest\;score$, responses scoring 65% and above were classified as having good TMP, and otherwise were classified as poor (Chanie et al., 2020).

Independent variables were selected based on a literature review that was considered to be factors associated with TMP and includes socio-demographic factors (age, sex, marital status, educational status, work experience, type of profession, monthly salary, having a child of < 18 years, type of institution, and work unit), job satisfaction factors (physical work environment, co-worker relationship, workload, performance appraisal, organizational policy and strategy, compensation and benefit, work autonomy, supervisor support, professional development, and recognition and reward), and personal factors (socialization and attitude of employees toward time and planning).

### Variable definition

#### Employees' attitude toward time

This variable shows the likelihood of employees' intention on effective time management utilization. It was measured by three items on a 5-point Likert scale. If the participant's response score is above the mean value of the sum score, it is represented as a good attitude, and otherwise, it is represented as a poor attitude (Abdulwadod, 2017).

#### Managers' attitude toward time

This variable indicates the managers' outlook on time management. It was measured using five items on a 5-point Likert scale. If the participant's response scored above the mean value, it indicated that they had a good attitude, and otherwise poor attitude (Abdulwadod, 2017).

#### Socialization

This variable states the level of participation in social activity of employees. It was measured by three items on a 5-point Likert scale. Participants who had scored more than the mean score of the responses to the items were considered to be very socially engaged, and otherwise considered to be poorly engaged (Meiring, 2017; Sayari et al., 2017).

#### Job satisfaction

This variable refers to the extent of positive or negative views of workers about their jobs. It was measured using 38 items, each having a 5-point Likert scale ranging from 1 (strongly dissatisfied) to 5 (strongly satisfied) using Minnesota satisfaction measurements (Weiss et al., 1967).

#### Dimensions of satisfaction

For each factor in the domain, the sum score of each item having values above the mean of the sum scores was considered satisfied and unsatisfied if it was below or equal to the mean of the sum scores (Geleto et al., 2015; Gedif et al., 2018; Temesgen et al., 2018).

#### Health service planning

This variable describes setting goals, scheduling, and outlining tasks. It was measured by four items on a 5-point Likert scale. The scores above the mean value were labeled as having a good plan and those with scores below and equal to the mean value were labeled as having a poor plan (Chanie et al., 2020).

### Data collection tool and procedure

A structured, pretested, and self-administered questionnaire was adapted from the literature for data collection (Qteat and Sayej, 2014; Owusu-Nketia, 2018; Asiedu, 2019; Nigussie and Injibara, 2019; Chanie et al., 2020). The survey was conducted face to face by using a printed questionnaire. The tool consisted of four sections: socio demographic factors (Section I), job satisfaction factors (Section II), personal factors (Section III), and TMP measurement items (Section IV). Data were collected by five trained diploma nurses and supervised by three B.Sc. nurses. The recruited nurses were trained on data collection procedures for 2 days. A pretest was performed at Woldia General Hospital and Kallu Speciality Clinic by taking 5% of the sample.

### Data quality control

A pretested questionnaire, trained data collectors and supervisors, and software used during the study ensure the quality of the data. Cronbach's alpha was used to check the validity of the tool, and each item was scored above 0.7. In addition, public health research experts reviewed the tools.

### Data processing and analysis

Data were checked for completeness, coded, and entered in the EPI-DATA version 4.6 and then exported to SPSS version 25. Frequencies, cross-tabulations, and tables were used for data presentation. The bivariable and multivariable logistic regression models were run separately for public and private hospitals after dichotomizing the outcome variable. To assess the association between the different predictor variables with the dependent variable, the first bivariable relationships between each independent variable and the outcome variable were investigated by using a binary logistic regression model. Those independent variables with a *p*-value of < 0.2 at the bivariable level were included in a multivariable analysis to control for possible confounding factors. A *p*-value of < 0.05 and a 95% confidence interval were used to declare a statistically significant association. The model fitness was assessed by Hosmer and Lemeshow (0.078 in public hospitals and 0.071 in private hospitals), and the models were reported to be fit. The collinearity diagnostic test was conducted to check for collinearity between independent variables, and the highest VIF was reported as 2.74 for the variable age. This indicates that multicollinearity was not an issue for this study.

## Results

A total of 615 participants responded to the survey and had a response rate of 93.2%. Among them, 409 and 206 samples were from public and private hospitals, respectively. Consequently, 60.2 and 58.7% of men worked in public and private hospitals, respectively. The median age (IQR) of the samples in public and private hospitals was 29 ± 4 and 29 ± 4.25 years, respectively. The median monthly salary (IQR) of the professionals in public and private hospitals was 7,071 ± 1,985 and 7,950 ± 4,050 ETB, respectively.

Almost one-fifth (20.4%) of the respondents in private hospitals had a poor attitude toward time. Moreover, 25.2 and 35.9% of respondents were found to be highly socially active in public and private hospitals, respectively. Nearly, 56.2 and 52.4% of the respondents had a good plan in public and private hospitals, respectively (Table 1).

**Table 1:** Sociodemographic characteristics of health professionals working in public and private hospitals in Dessie City, Ethiopia, 2021.

**Variable**	**Category**	**Private hospitals (*n* = 206)**	**Public hospitals (*n* = 409)**	**Overall (*n* = 615)**
		**Frequency (%)**	**Frequency (%)**	**Frequency (%)**
Sex	Male	124 (60.2)	240 (58.7)	364 (59.2)
	Female	82 (39.8)	169 (41.3)	251 (40.8)
Age category	20–24 years	50 (24.3)	43 (10.5)	93 (15.1)
	25–29 years	66 (32.0)	185 (45.2)	251 (40.8)
	30–34 years	43 (20.9)	101 (24.7)	144 (23.4)
	Above 35 years	47 (22.8)	80 (19.6)	127 (20.7)
Marital status	Unmarried	103 (50.0)	157 (38.4)	260 (42.3)
	Married	103 (50.0)	252 (61.6)	355 (57.7)
Educational level	Diploma	63 (30.6)	67 (16.4)	130 (21.1)
	Degree and above	143 (69.4)	342 (83.6)	485 (78.9)
Work experience in years	0–5 years	155 (75.3)	227 (55.5)	382 (62.2)
	6–10 years	40 (19.4)	138 (33.7)	178 (28.9)
	Above 10 years	11 (5.3)	44 (10.8)	55 (8.9)
Living condition	With family	123 (59.7)	293 (71.6)	416 (67.6)
	Without family	83 (40.3)	116 (28.4)	199 (32.4)
Profession	Nurse	82 (39.8)	254 (62.1)	336 (54.7)
	Doctor	50 (24.3)	57 (13.9)	107 (17.4)
	Laboratory professionals	26 (12.6)	37 (9.2)	63 (10.2)
	Pharmacists	23 (11.2)	38 (9.3)	61 (9.9)
	Anesthesiologists and Radiologists	25 (12.1)	23 (5.5)	48 (7.8)
Monthly salary	below 6,193 ETB	72 (35.0)	124 (30.3)	196 (31.9)
	6,193–8,017 ETB	58 (28.2)	165 (40.3)	223 (36.3)
	>8,017 ETB	76 (36.8)	120 (29.4%)	196 (31.9)
Working unit	Outpatient	77 (37.4)	202 (49.4)	279 (45.4)
	Inpatient	129 (62.6)	207 (50.6)	336(54.6)
Having < 18 years old child	Yes	83 (40.3)	183 (44.7)	266 (43.3)
	No	123 (59.7)	226 (55.3)	349 (56.7)

n, sample size; %, percentage.

### Job satisfaction factors

Nearly, 88.8% of health professionals were satisfied with coworker relationships in public hospitals, compared with 91.3% of health professionals in private hospitals.

On the other hand, 86.1, 80, 80.2, and 63.1% of health professionals in public hospitals were reported to have been dissatisfied with compensation and benefits, professional development, recognition and reward, and workload, respectively, compared to 56.3, 73.8, 71.8, and 63.6%, respectively, in private hospitals (Table 2).

**Table 2:** Job satisfaction-related factors among health professionals working in public and private hospitals in Dessie City, Ethiopia, 2021.

**Variable**	**Category**	**Private hospitals (*n* = 206)**	**Public hospitals (*n* = 409)**	**Overall (*n* = 615)**
		**Frequency (%)**	**Frequency (%)**	
Organizational policy and strategy	Unsatisfied	63 (30.6)	271 (66.3)	334 (54.3)
	Satisfied	143 (69.4)	138 (33.7)	281 (45.7)
Performance appraisal	Unsatisfied	57 (27.7)	237 (57.9)	294 (47.8)
	Satisfied	149 (72.3)	172 (42.1)	321 (52.2)
Compensation and benefit	Unsatisfied	116 (56.3)	352 (86.1)	468 (76.1)
	Satisfied	90 (43.7)	57 (13.9)	147 (23.9)
Physical working environment	Unsatisfied	78 (37.9)	295 (72.1)	373 (60.7)
	Satisfied	128 (62.1)	114 (27.9)	242 (39.3)
Professional development	Unsatisfied	152 (73.8)	327 (80.0)	479 (77.9)
	Satisfied	54 (26.2)	82 (20.0)	136 (22.1)
Supervisor support	Unsatisfied	51 (24.8)	235 (57.5)	286 (46.5)
	Satisfied	155 (75.2)	174 (42.5)	329 (53.5)
Work autonomy	Unsatisfied	54 (26.2)	186 (45.5)	240 (39.0)
	Satisfied	152 (73.8)	223 (54.5)	375 (61.0)
Workload	Low	131 (63.6)	258 (63.1)	389 (63.3)
	High	75 (36.4)	151 (36.9)	226 (36.7)
Recognition and reward	Unsatisfied	148 (71.8)	328 (80.2)	476 (77.4)
	Satisfied	58 (28.2)	81 (19.8)	139 (22.6)
Co-worker relationship	Unsatisfied	18 (8.7)	46 (11.2)	64 (10.4)
	Satisfied	188 (91.3)	363 (88.8)	551 (89.6)

n, sample size; %, percentage.

### Magnitude of time management practice

The general good time management practice among health professionals in both public and private hospitals was 57.1% (95% CI:53.0–60.8%). However, the magnitude differed between public [50.1% (95% CI:45.5–54.7%)] and private hospitals [70.9% (95% CI: 63.5–76.7%)].

### Associated factors of time management practice in public and private hospitals in Dessie City

In a separate multivariable logistic regression analysis of public and private hospitals, eight variables, four in public hospitals and four in private hospitals, were found to be statistically significant. In public hospitals, health professionals who were satisfied with organizational policy and strategy had 2.34 times higher odds of good TMP than unsatisfied respondents (AOR: 2.34, 95% CI: (1.25–4.36). Health professionals satisfied with performance appraisal had 1.95 times higher odds of good TMP compared to their counterparts (AOR: 1.95, 95% CI: 1.13–3.36).

The odds of good TMP were higher among respondents who were satisfied with work autonomy (AOR: 1.92, 95% CI: 1.12–3.27) in public and private hospitals (AOR: 7.10, 95% CI: 1.94–25.95). Employees who had a good attitude toward time had 2.12 times higher odds of TMP in public hospitals (AOR: 2.12, 95% CI: 1.26–3.58).

In private hospitals, living with a family increased the odds of good TMP by 5.39 times (AOR: 5.39, 95% CI: 1.84–15.77) compared to their counterparts. The odds of good TMP in private hospitals among respondents who were satisfied with compensation and benefits was 7.83 times higher than that in unsatisfied groups (AOR: 7.83, 95% CI: 1.97–31.16). Similarly, health professionals in private hospitals who planned better had 3.42 times higher odds of TMP than their counterparts (AOR: 3.42, 95% CI: 1.15–10.13; Table 3).

**Table 3:** Bivariable and multivariable logistic regression analyses of factors associated with TMP among health professionals working in public and private hospitals in Dessie City, Ethiopia, 2021 (*n* = 615).

**Variable**	**Category**	**Public hospitals (n** = **409)**				**Private hospitals (n** = **206)**			
		**TMP**		**COR (95% CI)**	**AOR (95% CI)**	**TMP**		**COR (95% CI)**	**AOR (95% CI)**
		**Good**	**Poor**			**Good**	**Poor**		
Sex of participants	Male	116	124	0.84 (0.57–1.25)	1.11 (0.65–1.89)	94	30	1.81 (0.98–3.23)	1.34 (0.44–4.05)
	Female	89	80	1	1	52	30	1	1
Age category	20–24 years	26	17	1	1	27	23	1	1
	25–29 years	91	94	0.63 (0.32–1.24)	1.19 (0.50–2.84)	44	22	1.70 (0.80–3.63)^*^	1.71 (0.47–6.22)
	30–34 years	40	61	0.43 (0.21–0.89)	0.60 (0.22–1.66)	39	4	8.31 (2.58–26.75)	5.15 (0.83–32.12)
	above 35 years	48	32	0.98 (0.46–2.09)	0.86 (0.30–2.48)	36	11	2.79 (1.16–6.69)^*^	1.25 (0.13–12.48)
Marital status	Unmarried	70	87	1	1	64	39	1	1
	Married	135	117	1.43 (0.96–2.1)	1.70 (0.88–3.28)	82	21	2.38 (1.28–4.44)^*^	0.85 (0.20–3.70)
Educational level	Diploma	36	31	1	1	39	24	1	1
	Degree and above	169	173	0.84 (0.50–1.42)	0.56 (0.29–1.08)	107	36	1.83 (0.97–3.45)	2.06 (0.55–7.77)
Living condition	With family	158	135	1.72 (1.11–2.66)^*^	1.90 (0.99–3.66)	97	26	2.59 (1.4–4.79)^*^	**5.39 (1.84–15.77)** ^*^
	Without family	47	69	1	1	49	34	1	1
Profession	Nurse	138	116	1	1	47	35	1	1
	Doctor	32	25	1.08 (0.60–1.92)	1.25 (0.62–2.50)	45	5	6.70 (2.41–18.63)^**^	1.00 (0.10–9.42)
	Laboratory professionals	15	22	0.57 (0.28–1.15)	0.45 (0.19–1.10)	15	11	1.02 (0.42–2.48)	2.32 (0.44–12.30)
	Pharmacists	14	24	0.49 (0.24–0.99)^*^	0.65 (0.27–1.57)	16	7	1.70 (0.63–4.58)	0.83 (0.15–4.61)
	Anesthesiologists and Radiologists	6	17	0.29 (0.11–0.78)^*^	0.33 (0.10–1.03)	23	2	8.56 (1.89–38.76)^*^	1.42 (0.17–11.62)
Working unit	Outpatient unit	110	92	1	1	50	27	1	1
	Inpatient unit	95	112	0.71 (0.48–1.05)	1.30 (0.80–2.34)	96	33	1.57 (0.85–2.99)	2.07 (0.53–8.00)
Monthly salary	Below 6,193 ETB	–	–	–	–	43	29	1	1
	6,193–8,017 ETB	–	–	–	–	39	19	1.38 (0.67–2,85)	0.86 (0.23–3.29)
	>8,017 ETB	–	–	–	–	64	12	3.60 (1.66–7.80)^**^	0.66 (0.07–5.86)
Having < 18 years old child	Yes	–	–	–	–	68	15	1	1
	No	-	–	–	–	78	45	0.38 (0.20–0.75)^*^	0.90 (0.18–4.50)
Organizational policy and strategy	Unsatisfied	108	163	1	1	28	35	1	1
	Satisfied	97	41	3.57 (2.30–5.54)^**^	**2.34 (1.25–4.36)** ^*^	118	25	5.90 (3.06–11.39)^**^	1.73 (0.52–5.74)
Performance appraisal	Unsatisfied	94	143	1	1	23	34	1	1
	Satisfied	111	61	2.77 (1.84–2.16)^**^	**1.95 (1.13–3.36)** ^*^	123	26	6.99 (3.55–13.77)	1.82 (0.50–6.59)
Compensation and benefit	Unsatisfied	167	185	1	1	61	55	1	1
	Satisfied	38	19	2.22 (1.23–3.99)	0.68 (0.31–1.49)	85	5	15.33 (3.79–40.55)^**^	**7.83 (1.97–31.16)** ^*^
Physical working environment	Unsatisfied	142	153	1	1	62	16	1	1
	Satisfied	63	51	1.33 (0.86–2.05)	0.81 (0.44–1.50)	84	44	0.49 (0.26–0.95)^*^	0.98 (0.28–3.40)
Professional development	Unsatisfied	152	175	1	1	–	–	–	–
	Satisfied	53	29	2.10 (1.27–3.48)^*^	1.33 (0.67–2.65)	–	–	–	–
Supervisor support	Unsatisfied	107	128	1	1	23	28	1	1
	Satisfied	98	76	1.54 (1.04–2.29)^*^	0.58 (0.32–1.06)	123	32	4.68 (2.38–9.19)^**^	1.56 (0.43–5.71)
Work autonomy	Unsatisfied	73	113	1	1	19	35	1	1
	Satisfied	132	91	2.25 (1.51–3.34)^**^	**1.92 (1.123.27)** ^*^	127	25	9.36 (4.63–18.92)^**^	**7.10 (1.94–25.95)** ^*^
Workload	Unsatisfied	–	–	–	–	98	33	1	1
	Satisfied	–	–	–	–	48	27	0.60 (0.32–1.12)^*^	0.76 (0.24–2.35)
Recognition and reward	Unsatisfied	155	173	1	1	100	48	1	1
	Satisfied	50	31	1.80 (1.09–2.96)	1.23 (0.58–2.64)	46	12	1.84 (0.89–3.79)	0.55 (0.16–1.87)
Co-worker relationship	Unsatisfied	–	–	–	–	7	11	1	1
	Satisfied	–	–	–	–	139	49	4.46 (1.64–12.14)^*^	2.16 (0.46–10.12)
Planning	Having a poor plan	74	105	1	1	54	44	1	1
	Having a good plan	131	99	1.88 (1.26–2.79)	1.53 (0.93–2.49)	92	16	4.69 (2.41–9.10)^**^	**3.42 (1.15–10.13)** ^*^
Employees' attitudes toward time	Poor attitude	47	88	1	1	24	18	1	1
	Good attitude	158	116	2.55 (1.66–3.91)^*^	**2.12 (1.26–3.58)** ^*^	122	42	2.18 (1.08–4.48)^*^	0.62 (0.19–1.99)
Socialization	Poorly socially active	163	143	1.66 (1.05–2.60)	1.37 (0.77–2.45)	–	–	–	–
	Highly socially active	42	61	1	1	–	–	–	–

COR, crude odd ratio; CI, confidence interval; AOR, adjusted odd ratio; 1, reference category.

^*^Significant at p < 0.05.

^**^Significant at p < 0.001.

–, Variable which is not a candidate for multivariable regression analysis.

n, sample size.

The bold value shows the significant variables indicated by the symbol^*^.

## Discussion

This is the first study that extensively explored the magnitude and factors of good TMP among health professionals in public and private hospitals in Dessie City. The magnitude of good TMP among health professionals in both public and private hospitals was 57.1% (95% CI: 53.0–60.8%). However, the magnitude differed between public [50.1% (95% CI: 45.5–4.7%)] and private hospitals [70.9% (95% CI: 63.5–76.7%)]. It is reported that TMP was 20.8% higher in private hospitals than in public hospitals. The magnitude of TMP in public hospitals was in line with a study conducted in Ghana (Asiedu, 2019). This consistency may be attributed to the selection of mixed health professionals rather than single professionals. However, the magnitude of TMP was lower than in studies conducted in Palestine (Qteat and Sayej, 2014), the United Arab Emirates (Al Khatib, 2014), India (Kulkarni, 2020), and Northern Gondar, Ethiopia (Chanie et al., 2020). This difference might be due to the infrastructure of health institutions, the study area, the sample size, and the characteristics of the study participants. Our study was also lower in connection with the magnitude of TMP than a study conducted among senior nurses in Iran (Ziapour et al., 2015). This difference could be attributed to the homogeneity and educational level of study participants, and the majority of the study participants in Iran had a bachelor's degree and above (Ziapour et al., 2015). However, the TMP in this study was higher than in a study conducted in Dire Dawa, Ethiopia (Nigussie and Injibara, 2019). This difference could be due to the recruitment of a small sample size as compared to the current study (Nigussie and Injibara, 2019).

In our study, the TMP was higher in private hospitals than in public hospitals. This variation might be due to poor job satisfaction for organizational policy, performance appraisal, and work autonomy dimensions in public hospitals as compared to private hospitals as identified in this study. If the satisfaction status of health professionals improved for the specified job satisfaction dimension, they would develop inspiration and keep their working time. It could be due to the poor control and management systems for health workers in public hospitals. This suggests that more attention is needed from health professionals in public hospitals to improve TMP. Additionally, unless early interventions are made, poor TMP practice in public hospitals will compromise the quality of services in their healthcare institutions.

Satisfaction with an organizational policy and strategy increased good TMP among health professionals working in public hospitals. This finding was in agreement with those of studies conducted in the Netherlands (Claessens et al., 2007), Egypt (Elsabahy et al., 2015), and Northern Gondar, Ethiopia (Chanie et al., 2020). This similarity could be justified because the health professionals were satisfied with the policy and strategy of their organization and may have a strong bond with their institution and can help them develop trust, ownership, and punctuality. This finding suggests that organizational policy development and design should consider the satisfaction level of health professionals to maximize TMP.

In the same vein, in public hospitals, being satisfied with the organization's performance appraisal system increased the odds of a good TMP. This finding was consistent with that of a study conducted in North Gondar, Ethiopia (Chanie et al., 2020), and it might be due to a similarity in infrastructure, type of participants, and the study setting. This association was also supported by Locke's discrepancy satisfaction theory, which stated that “the fewer discrepancies between the job outcomes an employee obtains and what is desired for him, the more likely to be satisfied” (Jiang et al., 2012). The theory of Adam's equity was also shared with Locke's discrepancy satisfaction theory which stated that “if there is equity in the outcome to input ratio of an employee with other employees, the more likely to be satisfied” (Thiagaraj and Thangaswamy, 2017). This finding should be considered during the preparation stage of the performance evaluation methods, and the tools need to be updated in a way to escalate health professionals' satisfaction level and ultimately foster TMP.

Moreover, health professionals who had a good attitude toward time had increased odds of a good TMP in public hospitals. This finding was supported by the Pickle Jar Theory of time management, which stated that “having a good attitude to manage distractions is the best way to manage our time, which in turn leads to goal achievement” (Wright, 2002). This finding builds on the existing body of evidence indicating that a good attitude toward time management would be a keystone to improving TMP (Yimer et al., 2023).

Furthermore, work autonomy significantly increased good TMP in both public and private hospitals. However, the strength of association is more pronounced in private hospitals. This finding was in agreement with the Herzberg motivation-hygiene theory, which stated that “the more employees are motivated by freedom of responsibility and other factors, the more they became satisfied” (Dugguh and Dennis, 2014). Therefore, if employees were autonomous in their work, they would be satisfied, accessible, and punctual to their jobs in the institutions they are being employed. This finding indicates that work autonomy is required in both study areas to augment TMP without compromising the quality of the health service.

In private hospitals, respondents who were satisfied with compensation and benefits showed increased good TMP than unsatisfied groups. This finding was supported by a job satisfaction theory of Adam's equity which stated that “if an employee perceives that he is not compensated fairly, he will not be happy and so slow the pace of performance” (Thiagaraj and Thangaswamy, 2017). Therefore, organizations must revise the compensation and benefit mechanisms to satisfy health professionals.

Similarly, in private hospitals, health professionals who lived with their families had higher odds of a good TMP. This finding was supported by a study conducted in the US (Halbesleben et al., 2009), which showed that family assistance could reduce their workload. This could be because healthcare professionals, who were supported by their families, could maintain their time for their work and would enable them to be punctual and committed at work. This could ultimately lead to improvement in individual performance and organizational success.

In private hospitals, having good planning significantly increases good TMP. This finding was in line with the Pareto theory of time management, which stated that “there is not enough time to do everything, but there is time to do urgent and important things.” The central idea of this theory shows the prioritization of tasks, which is one of the pillars of planning (Reh, 2013). If health professionals had a good plan, they would try to exercise their activities in accordance with their plan, which directly maximizes their TMP. This implies that proper planning plays an essential role in improving TMP. Therefore, health professionals must place special emphasis on health service planning to facilitate tasks effectively and use their time appropriately.

### Strengths and limitations of the study

A structured self-administered questionnaire has been used as a data collection tool. However, the responses of the participants to their practice may be different from what they practiced in real time. This study did not use the qualitative study method and also did not include primary health facilities. However, this study extensively addressed TMP by incorporating variables that were not addressed in previous studies such as socialization, work autonomy, workload, employees' attitudes toward time, profession, living conditions, co-worker relationships, professional development, and working unit. Furthermore, the novelty of this study lies in its comparison of TMP between public and private hospitals. Additionally, we did not study TMP separately in the category of specific healthcare professionals, which could influence a clear picture of the relationship between TMP and the profession so that great caution is needed during interpretation and application.

## Conclusion

The overall good TMP of public and private hospitals in Dessie City was low. However, the magnitude of a good TMP was higher in private hospitals than in public hospitals. Satisfaction with organizational policy and strategy, work autonomy, performance appraisal, and the good attitude of employees toward time significantly increased TMP in public hospitals, while living with families, good planning, satisfaction with compensation and benefits, and work autonomy were the factors that could influence TMP in private hospitals. The study enables health professionals working in public hospitals to take lessons from private hospitals to maximize time management practices in their institutions. More studies are required to address the problem extensively through the incorporation of observational and follow-up studies supported by qualitative methods focusing on primary health facilities and within a specific profession. Moreover, research focusing on work autonomy is also needed in the study area.

## Data availability statement

The original contributions presented in the study are included in the article/supplementary material, further inquiries can be directed to the corresponding author.

## Ethics statement

The studies involving humans were approved by the Institutional Review Board (IRB) of the University of Gondar. The studies were conducted in accordance with the local legislation and institutional requirements. The participants provided their written informed consent to participate in this study.

## Author contributions

AY: Conceptualization, Data curation, Formal analysis, Funding acquisition, Investigation, Methodology, Project administration, Resources, Software, Supervision, Validation, Visualization, Writing – original draft, Writing – review & editing. AF: Methodology, Supervision, Validation, Visualization, Writing – original draft, Writing – review & editing. LY: Visualization, Writing – original draft, Writing – review & editing. MAh: Methodology, Supervision, Visualization, Writing – original draft, Writing – review & editing. SS: Writing – original draft, Writing – review & editing. MAd: Writing – original draft, Writing – review & editing. SM: Supervision, Visualization, Writing – original draft, Writing – review & editing. MM: Writing – original draft, Writing – review & editing. NS: Writing – original draft, Writing – review & editing. NM: Writing – original draft, Writing – review & editing. DA: Writing – original draft, Writing – review & editing. ED: Writing – original draft, Writing – review & editing, Methodology, Supervision, Visualization. AK: Methodology, Supervision, Visualization, Writing – original draft, Writing – review & editing, Conceptualization, Data curation, Formal analysis, Software, Validation.
